# Risk and Protective Factors of Conflicts Between Hospitalized Older Adults and Their Family Members: Structural Equation Modeling (SEM)

**DOI:** 10.3390/bs15040405

**Published:** 2025-03-23

**Authors:** Ksenya Shulyaev, Anna Zisberg, Nurit Gur-Yaish

**Affiliations:** 1 The Minerva Center of Intersectionality in Aging, University of Haifa, Haifa 3103301, Israel; 2 Center of Research & Study of Aging, University of Haifa, Haifa 3103301, Israel; azisberg@univ.haifa.ac.il (A.Z.); nurit_g@oranim.ac (N.G.-Y.); 3 The Cheryl Spencer Department of Nursing, Faculty of Social Welfare and Health Science, University of Haifa, Haifa 3103301, Israel; 4 Oranim Academic College of Education, Kiryat Tivon 3600600, Israel

**Keywords:** older adults, hospitalization, family caregiver, family relationships, family conflict

## Abstract

Family relationships are important for the well-being of older adults, yet these relationships may involve ambivalence and/or conflict, particularly in high-stress scenarios such as hospitalization. This study aimed to identify factors predicting conflict between hospitalized older adults and family members, considering individual, social, and cultural factors. The sample comprised 573 cognitively intact older adults (65+) admitted to internal units in Israeli hospitals. Structural equation modeling (SEM) revealed that emotional support a decrease in conflict (β = −0.105, *p* = 0.007), while instrumental care (β = 0.146, *p* = 0.003), number of visitors (β = 0.125, *p* = 0.011), and the spouse being a primary caregiver (β = 0.159, *p* < 0.001) was associated with an increase in conflict. On the cultural level, being a Former Soviet Union (FSU) immigrant was a risk factor (β = 0.106, *p* = 0.016), while being an Israeli Arab had an indirect effect mediated by involvement in instrumental care and larger visitor numbers, which increased the risk for conflict (β = 0.087, *p* = 0.045). On the individual level, depressive symptoms increased conflict via emotional support (β = 0.01, *p* = 0.031), and independence in activities of daily living reduced conflict via lower instrumental care (β = −0.002, *p* = 0.003). These findings highlight the complex interplay of risk and protective factors in predicting conflict and highlight the role of social and cultural factors. Targeted interventions for spouses, caregivers providing instrumental support, and FSU immigrants may help reduce conflict during hospitalization.

## 1. Introduction

Family relationships are vital for health and well-being in old age (Carr & Utz, 2020), but these relationships sometimes involve ambivalence and/or conflict (Lüscher, 2002; Bengtson et al., 2002). While family ties can protect health by reducing stress (Umberson & Thomeer, 2020), strained family interactions can hamper autonomy and increase tension (Widmer et al., 2018). Considerable research has examined older adults’ family conflict in the community (Szydlik, 2008; Iveniuk et al., 2014) and the harmful effects of conflict between family members and terminally ill patients (Wilson et al., 2021). Less is known about the factors that predict conflict in the hospital setting.

Hospitalization is not uncommon for older adults (Beard et al., 2016). It is typically regarded as a stressful life event that challenges their coping skills and those of their family members (Rückholdt et al., 2017; da Rocha et al., 2022; Bridges et al., 2010). The impact of hospitalization on older adults and their family members extends to their relationships beyond the hospital stay, sometimes exacerbating existing tensions and leading to conflict (Hall, 1989). Although there are some intervention programs address communication between older adults and their caregivers in the community (Cheng & Zhang, 2020; Folder et al., 2024), our review of the literature discovered only one relevant program in the hospital (Akbari et al., 2015).

In the current study, we used Bronfenbrenner’s Human Ecological Theory (1979) to investigate the risk and protective factors of family conflict during older adults’ hospitalization (Bronfenbrenner, 1979). This theory offers a comprehensive framework for organizing the various levels of influence on conflict between families and older adults. According to Bronfenbrenner, individuals, with their distinct characteristics (individual-level factors), exist within nested and interactive systems of influence, ranging from the immediate environment (social-level factors) to broader societal structures (cultural-level factors). By accounting for these multi-level influences, the model enables researchers and practitioners to identify not only the immediate triggers of family conflict but also the broader systemic factors that may be targeted for intervention.

In Bronfenbrenner’s model, behavior cannot be fully understood without considering the specific context in which it occurs. According to this framework, it is crucial to evaluate whether evidence from one context, such as the community, aligns with evidence from another, such as the hospital. In the context of hospitalization, where heightened stress, uncertainty, and role shifts are common, this ecological lens provides a nuanced understanding of how individual, social, and cultural factors influence and interact in predicting conflict.

### 1.1. Individual Level

Even though we did not find other studies about conflict between older adults and family members in the hospital setting, findings from research in the community suggest it is influenced by individual factors. For example, research on conflict between family members and cancer patients found that female sex was a risk factor for conflict (Hamano et al., 2022), and old age (65+) was a protective factor (Kramer et al., 2010). Cognitive impairment was a significant predictor of conflict in community-dwelling older adults (Scharlach et al., 2006), with the patient’s cognitive status being related to caregiver burden (Lindt et al., 2020). Research in primary care confirmed that functional disability is associated with perceived family criticism (Seaburn et al., 2005), and the functional status of older adults is one of the strongest determinants of caregiver burden (Lindt et al., 2020; Rodríguez-González et al., 2021; Cartaxo et al., 2023). A qualitative study suggested that conflict arises when older adults feel a loss of independence, attempt to avoid burdening others, or lack trust in other supporters (Lindquist et al., 2018). Finally, research in primary care found that depression was associated with perceived family criticism (Seaburn et al., 2005), and a community study found that it was linked with lower satisfaction with caregivers’ communication in community-dwelling older adults (El Harsi et al., 2023).

### 1.2. Social Level

Family members are present in the hospital for an average of five hours per day (Gur-Yaish et al., 2012). A recent systematic review of caregiver burden risk factors in the community setting showed mixed results for the role of hours spent on caregiving. Some studies considered the hours spent on caregiving to be a determinant of caregiver burden, while others found that involvement in instrumental care, not the amount of time with older adults, was associated with caregiver burden (Lindt et al., 2020). During hospitalization of their older relatives, family members frequently provide emotional support, and, to a lesser degree, instrumental care (help with eating, bathing, and other activities of daily living (ADL)) (Gur-Yaish et al., 2012). Emotional support during hospitalization was found to have positive results in terms of older adults’ mental health (Gur-Yaish et al., 2013; Shulyaev et al., 2023, 2024). In a recent study of community-dwelling older adults, their receipt of emotional support, such as affective touch, predicted greater satisfaction with caregivers’ communication (El Harsi et al., 2023). Instrumental family care during a hospital stay has been related to higher levels of depression and anxiety among older adults, as well as lower recovery in ADL (Gur-Yaish et al., 2013; Shulyaev et al., 2023, 2024). Previous research on instrumental care in the community setting found that the intensity of care (i.e., the number of tasks and higher frequency of provision of instrumental care) was associated with a greater risk of caregiver burden (Cartaxo et al., 2023).

Informal caregiving typically encompasses a network of caregivers working collaboratively. These care networks for older adults are often complex and involve many individuals with varying levels of contribution and involvement and differing life impacts (Tang et al., 2018). Older adults have an average of two visitors during the hospital stay (Gur-Yaish et al., 2013). Greater numbers of visitors when there is a larger caregiving network can be a protective factor against caregiving burden (Tolkacheva et al., 2011). However, there is a need for effective coordination, and this can be challenging, as different caregivers may have varying approaches and communication styles, potentially leading to confusion for the older adult (Tang et al., 2018).

Previous research found that older adults are primarily accompanied in hospitals by adult children and spouses (Gur-Yaish et al., 2012; Shulyaev et al., 2024). Research about the impact of the figure that provides help had mixed results. Two studies found that adult children were at greater risk of caregiving burden (Chappell et al., 2014; Rigby et al., 2019), while another reported an absence of differences in burden between children and spouses (Savundranayagam et al., 2011). Still other work argued that spouse–caregivers had a greater risk of adverse physical and psychological outcomes (Schulz & Eden, 2016).

### 1.3. Cultural Level

The culture of patients and their families includes varying expectations and familial roles, and it influences how caregiving responsibilities are perceived and managed (Halperin, 2013; Youn et al., 1999; Katz, 2009; Miyawaki, 2016). Studies of family conflicts among patients with cancer and diabetes in the community setting found that ethno-cultural groups differed in the probability of conflict. Specifically, ethnic minority status was a significant risk factor for conflicts (Kramer et al., 2010; Fox et al., 2020). Hospitalization brings together two cultural contexts of care: the personal and the organizational. This might pose extra challenges for patients and caregivers from minority groups (Degrie et al., 2017; Popper-Giveon, 2019). Additionally, the stressful aspects of hospitalization might require different ethno-cultural groups to use different coping strategies (Knight & Sayegh, 2010). Previous research on Israeli older adults found that the cultural background can affect family caregiving during acute hospitalization. Patients from the Former Soviet Union (FSU) are less likely to have informal caregiver visits, family visits are shorter, and families provide less supervision of medical care (Auslander, 2011). In contrast, caregivers from Israeli Arab society are more likely to stay in hospital during the night (Shulyaev et al., 2020). Taken together, these findings suggest the need to investigate the role of ethno-cultural groups in family conflict in the unique context of the hospital.

### 1.4. Interactions Within and Between Levels

Bronfenbrenner’s Human Ecological Theory emphasizes the importance of interactions occurring within each structural level (individual, social, cultural), as well as between levels (Härkönen, 2007). For example, dependence in performing ADL, an individual characteristic, is often associated with depression in older adults (Shulyaev et al., 2024; Gur-Yaish et al., 2012). In addition, emotional and instrumental family support, a social factor, during hospitalization influences the mental and physical status of older adults (Gur-Yaish et al., 2013; Shulyaev et al., 2023, 2024), and the culture of patients and their families shape patterns of family caregiving during hospitalization (Shulyaev et al., 2020). However, to the best of our knowledge, no study has examined how these factors interact in predicting conflict between older adults and their families during acute hospitalization.

In summary, there is a clear understanding of the importance of family relationships for older adults and the impact of family conflict on their well-being. Research has also established that hospitalized older adults are commonly accompanied by family members. However, the risk and protective factors contributing to conflict between hospitalized older adults and their family members have not been studied. The proposed ecological framework allows a holistic examination of how the various factors contribute to conflict.

### 1.5. Study Hypotheses

We aimed to identify predictors of conflict between hospitalized older adults and family members across individual, social, and cultural levels. Based on the literature and our theoretical framework, we developed two hypotheses:

**H1.**  *Individual (sex, functional and cognitive status, depressive symptoms), social (emotional and instrumental support from family, number of visitors, duration of family visit, relationship to caregiver), and cultural factors (belonging to Israeli Arab or FSU-immigrant cultural groups) will directly influence the risk of conflict between older adults and their family members during acute hospitalization*.

**H2.**  *Individual, social, and cultural factors will not only have independent effects but will also interact in influencing conflict. Specifically, individual (e.g., functional status, depressive symptoms) and cultural factors (e.g., belonging to Israeli Arab or FSU-immigrant groups) will shape conflict indirectly through their impact on social factors, such as family support, number of visitors, and family involvement in care.*

## 2. Materials and Methods

### 2.1. Study Design and Setting

This study analyzed data collected in a multi-center prospective cross-sectional observational study, ‘Hospitalization Process Effects on Mobilization Outcomes and Recovery’ (HoPE-MOR), that tested the association between personal risk factors, hospitalization care processes, and level of in-hospital mobility (see Gur-Yaish et al., 2021). The original study was conducted in four hospitals in northern Israel between 2018 and 2021. The hospitals serve diverse populations, and each has unique characteristics related to the surrounding urban/rural communities. The original sample included cognitively intact Hebrew-, Russian-, and Arabic-speaking older adults (≥65 years) acutely hospitalized in internal units. Exclusion criteria were acute stroke or coma, mechanical ventilation, admission to end-of-life care, cognitive impairments, or total dependency in mobility prior to admission. Hospitalized patients were followed prospectively from their admission to an internal medical unit to the end of their hospital stay and then at one-month post-discharge.

Out of the original sample of 762 older patients with full baseline data, 181 (28.5%) were excluded from the current analysis due to uncomplete data (92 lacked data on study variables, such as conflict with family member or receipt of family support; 38 refused to continue; 17 had a short hospital stay; 14 were discharged without completing the questionnaire; 11 were excluded because of severe cognitive decline; 3 died; 2 had no family visits; 2 were isolated because of infection; 2 were moved to another unit). Eight were excluded because they were new immigrants not from the FSU. No statistically significant differences were observed in age and sex ((F_(760,1_) = 1.76, *p* = 0.19; F_(760,1_) = 0.28, *p* = 0.59) or between participants included in and excluded from this data analysis and the original study sample. However, excluded potential participants had lower average cognitive and functional status (F_(751,1_) = 10.27, *p* = 0.001; F_(752,1_) = 6.35, *p* = 0.012).

### 2.2. Instruments and Measures

#### 2.2.1. Conflict Between Hospitalized Older Adults and Family Members

Conflict between older adults and their family members during hospitalization was measured at discharge using questions about the relationship with the primary caregiver (family member who provided the most support and care) during hospitalization. We adapted the Conflict Scale developed and validated by Silverstein et al. (2010) by adding “during hospitalization” to all items. The questionnaire included: “How much conflict, tension, or disagreement do you feel there is between you and this family member during hospitalization?”; “How much do you feel this family member is critical of you during hospitalization?”; “How much does this family member argue with you during hospitalization?” Participants rated their responses on a Likert scale ranging from 1 (not at all) to 6 (very much) (Katz, 2009). The questionnaire was translated to Hebrew, Arabic, and Russian. The accuracy of the translations was ensured through back-translation methods and extensive pretesting of the instrument.

The scale showed good reliability for this study (α = 0.77). The average score was not normally distributed (Skewness 2.632 (0.102); Kurtosis 7.743 (0.203). Therefore, we dichotomized the variable to differentiate between those who reported no conflict (score = 1) and those who reported any level of conflict (score > 1).

#### 2.2.2. Individual Characteristics of Older Adults

Independence in ADL was estimated using self-reports of 10 items from the Modified Barthel Index for Activities of Daily Living (BADL) (Shah et al., 1989).

Cognitive function was assessed using the Telephone Version of the Mini-Mental State Examination (MMSE-T). The MMSE-T is a brief measure of cognition assessing domains of orientation, attention, concentration, memory, and language (Roccaforte et al., 1992).

Depression symptoms were assessed using seven questions on depression symptoms from the Hospital Anxiety and Depression Scale (HADS) (Zigmond & Snaith, 1983). The reliability of the Depression subscale was good (α = 0.80).

Background characteristics included sex (male/female), native language, country of birth, and religion. All variables were self-reported by patients within the first 24 h of hospital admission.

#### 2.2.3. Characteristics of Caregiving During Hospitalization

All characteristics of caregiving during hospitalization were assessed daily from the second day of hospitalization until discharge (or up to 7 days).

Family involvement in caregiving during hospitalization was assessed with two subscales of the Informal Caregiving for Hospitalized Older Adults (ICHOA) scale: instrumental care (e.g., help with eating or grooming) and emotional support (e.g., comforting patients when sad or helping them get used to the hospital) (Gur-Yaish et al., 2012). Each subscale includes three items evaluated on a Likert-type scale from 0 (did not receive any help) to 4 (received help all the time). We calculated the mean for each subscale and used this continuous variable in further analysis as a level of family involvement in this kind of support. For our study, the reliability of ICHOA scale items was good (α = 0.85 for instrumental care; α = 0.87 for emotional support).

The length of family members’ visits during the day was self-reported daily by patients and measured in hours. We then calculated the mean score of all days of hospitalization.

Number of visitors during the day was self-reported by patients daily. If the number of visitors was more than 10, it was reduced to 10. Then, we calculated the mean number of visitors on all days of hospitalization.

#### 2.2.4. Ethno-Cultural Group

Israeli society includes a variety of national, religious, secular, and ethnically unique population groups. We focused on Arabs, Jewish immigrants from the FSU, and Israeli-born or veteran immigrant Jews. Arabs and FSU immigrants comprise the largest minority groups in the Israeli older adult population (13% and 18.8%, respectively) (Brodsky et al., 2018).

Participants were divided into three ethno-cultural groups according to their self-identified native language (Hebrew, Arabic, or Russian), religion (Jews, Christians, or Muslims), country of birth (Israel, FSU, or other countries), and year of immigration (before or after 1989, with the former considered veteran immigrants) (Shulyaev et al., 2020). The three groups were (1) Israeli Jews: Israeli-born with Hebrew as a mother language and/or veteran immigrants arriving before 1989; (2) Israeli Arabs: Israeli-born with Arabic as a mother language and Christian, Muslim, or Druze religion; (3) FSU immigrants: immigrants from the FSU arriving after 1989 with Russian as their native language.

### 2.3. Statistical Analysis

We computed descriptive statistics for all study variables to summarize the sample characteristics. To examine the relationship between the study variables and conflict with family members during hospitalization, we conducted a one-way ANOVA for numeric variables and a chi-square test for categorical variables. We took a conservative approach and used a 0.10 threshold level in the univariate analysis to decide on variable inclusion in the structural equation modeling (SEM) predictors of conflict. All analyses were performed using IBM SPSS Statistics version 27. For structural equation modeling (SEM), we used IBM SPSS AMOS version 29.

## 3. Results

### 3.1. Participant Characteristics

A total of 573 older adults participated in the study. The mean age was 77.43 years (SD = 6.8), with a slight majority of males (55%). Participants had high cognitive and functional levels and comparatively low levels of severity of illness and comorbidities. The average education length was about 12 years (duration of secondary education in Israel), but the sample also included persons without education and with higher academic degrees. The level of religiosity and socioeconomic status were average. Family members who provided most of the support during hospitalization were children (49.4%) and spouses (36.6%). “Others” were grandchildren, spouses of children, siblings, and, in rare cases, other family members or friends. The average daily duration of a family visit was around five hours. Most participants were Israeli Jews (59.3%), around a quarter were FSU immigrants (24.2%), and the rest were Israeli Arabs (16.5%). Thirty percent of respondents (*N* = 170) reported some kind of conflict with family members during hospitalization. Table 1 contains a description of participants’ characteristics.

### 3.2. Association of Participants’ Characteristics with Conflict During Hospitalization

We analyzed differences between older adults who reported (*N* = 170) and did not report (*N* = 405) any kind of conflict with family members during hospitalization. Based on the literature, we included the following variables potentially associated with conflict: (1) on the individual level: sex, cognitive status (MMSET), level of independence in ADL, level of depressve symptoms (HADS); (2) on the social level: the kind of family member providing most ot the support during hospitalization, duration of family visits, number of visitors, involvement of family in instrumental care and emotional support during hospitalization (ICHOA); (3) on the cultural level: ethno-cultural group (FSU immigrants, Israeli Arabs, Israeli Jews).

#### 3.2.1. Individual Level

There were no significant differences between the two groups (conflict/no conflict) in sex and cognitive status. The only significant difference was in independence in ADL. Older adults reporting conflict were less independent (F_(1,571)_ = [7.281], *p* = 0.007, ɳ^2^ = 0.013). The difference in depressive symptoms at admission approached significance (F_(1,571)_ = [3.296], *p* = 0.070, ɳ^2^ = 0.006), with patients reporting conflicts tending to have more depressive symptoms at admission.

#### 3.2.2. Social Level

Patients reporting conflict had more visitors per day (F_(1,571)_ = [8.755], *p* = 0.003, ɳ^2^ = 0.015) and higher involvement of family in instrumental care (F_(1,571)_ = [6.567], *p* = 0.009, ɳ^2^ = 0.012). Differences in the level of emotional support provided approached significance (F_(1,571)_ = [3.642], *p* = 0.057, ɳ^2^ = 0.006), with a trend towards less emotional support reported by those experiencing conflict. There were no significant differences between groups in average duration of family visit, but a chi-square test of independence revealed differences based on the kind of family member who provided the most support and help during hospitalization (spouse, children, others): χ^2^ (2, *N* = 575) = 7.233, *p* = 0.027. More spouses and fewer children and others were in the group who reported conflict than in the group who did not.

#### 3.2.3. Cultural Level: Ethno-Cultural Group

The differences between the three ethno-cultural groups approached significance (χ^2^ (2, *N* = 573) = 5.370, *p* = 0.068). There were fewer Israeli Jews and more Israeli Arabs and immigrants from the FSU in the group who reported conflicts than in the group who did not. Table 1 contains a detailed description of the differences between groups.

### 3.3. SEM of Factors Predicting Conflict During Hospitalization

Factors associated or approximately associated with conflict that we included in the SEM analysis were independence in ADL, depressive symptoms, number of visitors, family involvement in instrumental care and emotional support, kind of family member providing the most support (binary variable: spouse/not spouse), and ethno-cultural group (two dummy variables: FSU immigrant (0/1), Israeli Arab (0/1)). Before including variables in the model, we assessed the SEM assumption of absence of multicollinearity. Tolerance values were all above 0.2 (range: 0.609–0.965), and the variance inflation factor (VIF) was below 2 (range: 1.036–1.641), indicating no significant multicollinearity.

**Figure 1:**
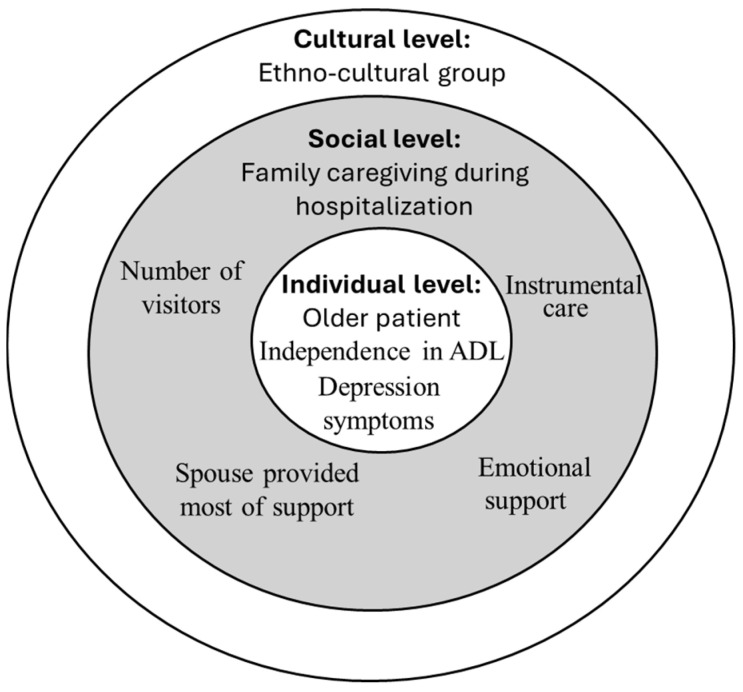
Ecological model of factors associated with conflict between older adults and family members during acute hospitalization. Note: This conceptual model was developed for this study.

SEM is used to justify the acceptance or rejection of a proposed hypothesis by analyzing the direct and indirect effects of mediators on the relationship of independent and dependent variables (Kumar & Upadhaya, 2017). The multivariate study conceptual model organized by ecological individual, social, and cultural levels is shown in Figure 1.

To better understand the interaction between factors associated with conflict between older adults and family members during hospitalization, we first tested the bivariate associations between variables and then included in to the model SEM pathways between significantly correlated relationships variables. The association between study variables is shown in Table 2.

**Figure 2:**
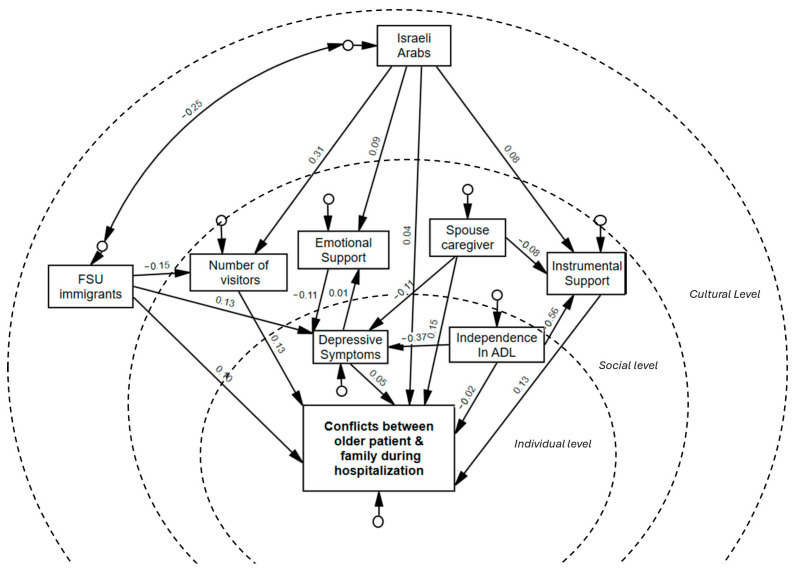
SEM of factors associated with conflict between older adults and family members during acute hospitalization. Note: Figure created by the authors based on study data.

In our SEM, we tested for multivariate normality, absence of missing data, and model fit indexes. The SEM analysis included 18 (9 observed and 9 non-observed) variables (see Figure 2). Overall, the default model demonstrated a good fit to the data, supported by most of the fit indices, including the RMSEA, SRMR, GFI, CFI, and NFI (Mueller & Hancock, 2011) (see Table 3).

The SEM analysis revealed significant relationships between the factors contributing to conflict between older patients and family members, with both direct and indirect pathways influencing these dynamics (see Table 4).

We observed several direct effects highlighting the impact of social-level factors. Specifically, emotional support was associated with a decrease in conflict probability (β = −0.105, 95% CI [−0.186, −0.027], *p* = 0.007). Instrumental care exhibited a positive relationship with conflict (β = 0.146, 95% CI [0.048, 0.248], *p* = 0.003), as did the number of visitors (β = 0.125, 95% CI [0.029, 0.215], *p* = 0.011). Notably, receiving most support from a spouse directly increased conflict levels (β = 0.159, 95% CI [0.074, 0.238], *p* < 0.001). No significant direct effect emerged for individual-level factors. Increased dependency in ADL (*p* = 0.659) and depression symptoms (*p* = 0.483) had no significant direct effect on conflict. On the cultural level, being an FSU immigrant was positively associated with conflict (β = 0.106, 95% CI [0.019, 0.193], *p* = 0.016), but being an Israeli Arab had no significant direct effect (*p* = 0.309).

Several indirect effects emerged, revealing key mediating pathways. Depressive symptoms influenced conflict indirectly through emotional support (β = 0.01, 95% CI [0.001, 0.024], *p* = 0.031). Independence in ADL was indirectly associated with lower conflict through its impact on instrumental care provided by family (β = −0.002, 95% CI [−0.004, −0.001], *p* = 0.003). Among Israeli Arabs, the relationship between cultural background and conflict was fully mediated through instrumental care (β = 0.014, 95% CI [0.001, 0.034], *p* = 0.04) and number of visitors (β = 0.048, 95% CI [0.011, 0.089], *p* = 0.011) as risk factors, and emotional support (β = −0.012, 95% CI [−0.026, −0.001], *p* = 0.011) as a protective factor. Even though one of the mediation paths involved the protective factor of emotional support, the total effect significantly predicted greater conflict with relatives during hospitalization among Israeli Arabs (β = 0.087, 95% CI [0.002, 0.175], *p* = 0.045). In contrast, among FSU immigrants, the number of visitors mediated negative effect on conflict (β = −0.02, 95% CI [−0.036, −0.004], *p* = 0.011). Similarly, among FSU immigrants, a lower number of visitors partially mediatednegative effect on conflict (β = −0.02, 95% CI [−0.036, −0.004], *p* = 0.011), but the total effect on conflict was significant and positive (β = 0.093, 95% CI [0.008, 0.179], *p* = 0.033). Additionally, receiving most support from a spouse indirectly reduced conflict through less involvement in instrumental care (β = −0.011, 95% CI [−0.024, −0.001], *p* = 0.017) and through lower depressive symptoms and higher emotional support (β = −0.001, 95% CI [−0.003, 0], *p* = 0.034). The total effect was positive; receiving most support from a spouse contributed positively to conflict (β = 0.142, 95% CI [0.059, 0.222], *p* = 0.001).

## 4. Discussion

We aimed to identify the factors predicting conflict between hospitalized older adults and their family members and the interplay between these factors using Bronfenbrenner’s Human Ecological Model (1979) (Bronfenbrenner, 1979). Our findings highlight the importance of social and cultural factors in shaping conflict during acute hospitalization, as well as the interplay between individual, social, and cultural factors.

### 4.1. H1: Individual, Social, and Cultural Factors Directly Affecting Conflict

We hypothesized that individual factors (sex, functional and cognitive status, depressive symptoms), social factors (emotional and instrumental support from family, duration of visits, number of visitors, relationship to caregiver), and cultural factors (Israeli Arabs, FSU immigrants) would directly influence the risk of conflict between older adults and their family members during acute hospitalization. Our hypothesis was partially confirmed; most social factors (except for visit duration) directly affected the likelihood of conflict. However, individual-level factors did not exert a direct influence. As for cultural factors, being an FSU immigrant directly predicted a higher likelihood of conflict, whereas being an Israeli Arab had no direct effect.

Four social-level predictors directly affected the probability of conflict; emotional support acted as a protective factor, while instrumental support, more support provided by spouse, and larger numbers of visitors were identified as risk factors.

The finding that emotional support was a protective factor against conflict between older adults and family members is not surprising. First, family members and hospital staff agree on the importance of family members’ emotional support (Gur-Yaish et al., 2021). Second, previous findings demonstrated the positive impact of emotional support on depressive symptoms among older adults (Gur-Yaish et al., 2013). Our findings extend these previous findings by suggesting that emotional support from close others in times of stress, like hospitalization, has a positive effect on the quality of the relationship.

The finding that instrumental support was a significant risk factor of conflict between older adults and family members is also not surprising. Previous studies have indicated the negative consequences of instrumental support at the individual level (Gur-Yaish et al., 2013). In addition, community-based studies have suggested that older adults’ loss of independence and fear of burdening others can lead to conflicts with family members (Lindquist et al., 2018). In the hospital setting, family members report that they are unsure about how to assist older adults with ADL in a hospital environment (Edwards et al., 2017). Taken together, these studies, along with the current investigation, highlight the challenge of providing instrumental support to an older relative without affecting the individual or the relationship. We found older adults who were accompanied by their spouses had an increased risk of conflict. Research in the community setting has suggested that the chronic illness of a partner is related to difficulties in communication, higher burnout, and less satisfaction for both partners (Shrout et al., 2024). It is reasonable to assume that hospitalization, with its additional stress and disruption of routines (Zisberg & Gur-Yaish, 2017), will intensify these difficulties and lead to conflict.

The final social predictor of conflict was the number of visitors. Even though studies from the community have found that a larger caregiving network can be a protective factor against caregiver burden (Tolkacheva et al., 2011), our study is in line with others suggesting that larger networks might lead to confusion for older adults (Tang et al., 2018). Moreover, in times of acute illness, visitors might disrupt older adults from concentrating on the task of healing, leading to more conflict.

On the cultural level, FSU immigrants reported more conflict with family members during hospitalization. FSU immigrants generally have fewer resources, greater financial burdens (Gorbatkin et al., 2021), higher disease prevalence rates (Rennert et al., 2002), lower life satisfaction (Katz, 2009), and higher levels of anxiety during hospitalization than Israeli Jews and Arabs (Zisberg, 2017). A possible explanation for the greater risk of conflict between older FSU immigrants and their family members during acute hospitalization, beyond the strain of immigration, is that cultural differences and generational gaps that may lead to misunderstandings and mismatched expectations of caregiving roles and responsibilities. The stress of hospitalization, compounded by language barriers and the pressure of navigating an unfamiliar healthcare system (for both older adult patients and their families), can exacerbate existing tensions and lead to conflict during critical moments of care.

### 4.2. H2: Individual, Social, and Cultural Factors Indirectly Affecting Conflict

We hypothesized that we would find interactions between factors at the individual, social, and cultural levels. Specifically, we expected individual- and cultural-level factors would indirectly affect conflict through social factors. This hypothesis was partially supported by the SEM analysis; depressive symptoms, functional status (independence in ADL), and belonging to the ethno-cultural group of Israeli Arabs were fully mediated by social-level factors.

While lower levels of independence in ADL and higher levels of depressive symptoms were correlated with a higher risk of conflict, these individual-level factors had no significant direct effect in the study model. However, both had indirect effects: depressive symptoms via lower emotional support, and lower independence in ADL via more involvement of family with instrumental care. It seems that it is not the individual-level factors per se but their consequences on caregiving patterns that affect the relationship quality.

Even though Israeli Arabs exhibited a slightly higher frequency of conflict with family members during hospitalization, being Arab did not directly affect the probability of conflict. Instead, it affected conflict indirectly through the interplay with social-level variables: having more visitors and greater involvement in instrumental care. These caregiving patterns are in line with the Arabic cultural tradition of respect for older adults and filial piety; these norms encourage involvement in care (Khalaila & Litwin, 2012), and the extension of family support networks to include larger families with more children, friends, and neighbors (Lowenstein & Katz, 2015). Paradoxically, in our study, these caregiving patterns resulted in more conflict between hospitalized older adults and their family members. The unique context of providing care during hospitalization might challenge the traditional patterns of older Arab patients and their family members.

It is important to note that some of the risk factors identified in community studies were not related to conflict in the present study. Patients’ cognitive status has been associated with conflict in other studies (Hamano et al., 2022; Scharlach et al., 2006) but was not significantly associated with conflict during hospitalization. In addition, the average duration of family visits was not associated with conflict in our study, even though other work has identified it as a risk factor in the community. Hospital visits are usually limited and might affect conflicts differently than visits in the daily routine of home care. These differences between risk and protective factors of conflict between older adults and their family members in the community and the hospital highlight the importance of Bronfenbrenner’s assertion that behavior cannot be fully understood without considering the specific context in which it occurs.

### 4.3. Limitations

This study is based on cross-sectional data, which prevents us from establishing causality. While we discuss certain variables as ‘risk’ or ‘protective’ factors, these terms should be interpreted in a correlational rather than a causal sense. Future research employing longitudinal or experimental designs is needed to confirm the directionality and causal nature of these associations. The limitation of our study is that it reduced conflict scale to a binary outcome which may lead to a loss of important information and need to be explored in future studies. Another significant limitation is that the data collection relied on older patients’ self-reporting, which can lead to social desirability bias and recall bias. Family members/caregivers were not included in the study, making the assessment of conflict one-sided. This bias is especially warranted regarding the reporting of conflicts. Studies about conflict between family members and older adults in the community suggested that the best way to estimate the risk of conflict is by assessing family relationships from multiple perspectives (Hamano et al., 2022). Given that conflict is a relational construct, the absence of family members’ perspectives may introduce bias, as patients may either understate or exaggerate tensions depending on their emotional state, dependency level, or expectations of caregiving. Future research could benefit from the triangulation of perspectives to provide a more comprehensive view by exploring family-level dynamics and including the viewpoint of family members as well. Another limitation is that, in the current study, the level of conflict was measured only at discharge. Even though the ecological model allowed us to map the relevant variables into the different layers, it provided a static picture of the predictors of conflict in the hospital. It is imperative to measure conflict after hospitalization as well, since it is logical to assume that hospitalization and its long-term effect take their toll on both older adults’ and family caregivers’ mental health and do not disappear at the time of discharge (Suitor et al., 2018; Widmer et al., 2018). Future studies might reveal whether hospitalization is the trigger of the conflict, and whether it extends to relationships beyond the hospital stay. Additionally, all hospital settings included in the study have uniform visiting policies, which limit our ability to assess their impact on the situation. Future research should include hospital settings with varied visiting policies to better understand their influence on conflicts between hospitalized older patients and their family members. Finally, the study’s generalizability may be restricted because the sample primarily consisted of highly functioning older adults, indicating a need for further evaluation in groups with more dependent older adults and other hospital settings.

## 5. Conclusions

The study identified key individual, social, and cultural predictors of conflict between older adult patients and their family members during acute hospitalization. The findings suggest the need to develop educational intervention programs for hospital staff to train them to identify families at higher risk of conflict during hospitalization. Additionally, the data on the risk and protective factors of family conflict could serve as the foundation for creating a specialized screening tool to detect older patients at risk of family conflict at admission to the hospital.

Our study’s insights into the interaction between individual characteristics, caregiving patterns, and cultural differences provide a basis for designing tailored intervention programs for visiting family members from high-risk groups. For instance, family members of older patients who are not independent in ADL require specific interventions to help them provide instrumental support in ways that minimize conflict and avoid negative side effects. In Israel, these intervention programs must be culturally adapted for Israeli Arab families and offered in Arabic, as this ethno-cultural group tends to be more involved in providing instrumental support, increasing the likelihood of conflict. Israeli Arab families may also benefit from educational interventions emphasizing the importance of limiting the number of visitors during hospitalization to reduce tensions between older adults and their family members. Although the importance of family support is widely recognized, it may still be necessary to consider restricting the number of visitors to reduce conflict and improve care outcomes.

For older patients with symptoms of depression, visiting family members should be trained to provide appropriate emotional support tailored to the patient’s age, depressive symptoms, and the stress of acute hospitalization. Such emotional support training should be culturally adapted for FSU-immigrant families and delivered in Russian. This is particularly important, as older patients from this group often present higher rates of depression, and a lack of emotional support increases the risk of conflict. In conclusion, the current study is the first to comprehensively examine the interplay of individual, social, and cultural factors in predicting conflict between older patients and their family during hospitalization, paving the way for targeted interventions to foster more harmonious relationships and reduce conflict during this critical period.

## Figures and Tables

**Table 1:** Participant characteristics (*N* = 573).

		Total (*N* = 573)	No Reported Conflict (*N* = 404)	Reported Conflict (*N* = 169)		Effect Size
		M ± SD	M ± SD	M ± SD	F	(ɳ^2^)
Cognitive status	MMSET (7–22)	18.8 ± 3	18.92 ± 2.8	18.5 ± 3.3	1.61	0.003
Independence in ADL at admission	Bartel 0–100	89.95 ± 17.6	91.23 ± 16	86.89 ± 20.7	7.28 **	0.012
Depression symptoms	HADS (0–21)	5.49 ± 4.4	5.27 ± 4.3	6 ± 4.5	3.30 ^a^	0.005
Daily duration of family visit	hours 0–16	5.12 ± 3.8	4.96 ± 3.8	5.52 ± 3.7	2.64	0.004
Number of visitors	0–10	3.47 ± 3.4	3.2 ± 2.9	4.11 ± 4.3	8.76 **	0.015
Family involvement in	ICHOA (0–4)					
Instrumental care		0.66 ± 1.2	0.57 ± 1.2	0.86 ± 1.3	6.87 **	0.022
Emotional support		3.07 ± 1.2	3.14 ± 1.2	2.93 ± 1.1	3.64 ^a^	0.006
		*N* (%)	*N* (%)	*N* (%)	*χ* ^2^	
Sex of patient	Male	314 (54.8%)	213 (52.7%)	101(59.8%)	2.39	
	Female	259 (45.2%)	191 (47.3%)	68 (40.2%)		
Family member providing most support	Spouse	210 (36.6%)	135 (33.4%)	75 (44.0%)	7.233 *	
	Child	283 (49.4%)	206 (51.0%)	77 (45.6%)		
	Other	80 (14.0%)	63 (15.6%)	17 (10.1%)		
Ethno-cultural group	Israeli Jews	339 (59.2%)	251 (62.1%)	88 (52.1%)	5.370 ^a^	
	FSU immigrants	139 (24.3%)	93 (23.0%)	46 (27.2%)		
	Israeli Arabs	95 (16.5%)	60 (14.9%)	35 (20.7%)		

^a^ *p* < 0.1; * *p* < 0.05; ** *p* < 0.01.

**Table 2:** Association between study variables (*N* = 573).

		1	2	3	4	5	6	7
Independence in ADL	1	1						
Depression symptoms	2	−0.382 **	1					
Instrumental care	3	−0.578 **	0.282 **	1				
Emotional support	4	−0.028	−0.092 *	0.095 *	1			
Number of visitors	5	−0.097 *	−0.046	0.097 *	0.023	1		
Spouse caregiver	6	0.149 **	−0.183 **	−0.162 **	0.044	−0.046	1	
Israeli Arabs	7	−0.112 **	0.036	0.145 **	0.086 *	0.350 **	−0.047	1
FSU immigrants	8	0.005	0.132 **	−0.057	0.006	−0.226 **	−0.090 *	−0.251 **

* *p* < 0.05; ** *p* < 0.01.

**Table 3:** Goodness-of-fit indices for the SEM of factors associated with conflict between older patients and family members during hospitalization.

	CMIN/DF	RMSEA	SRMR	GFI	AGFI	NFI	CFI
Recommended Values	<3	<0.06	<0.08	>0.90	>0.90	>0.90	>0.95
Default Model	2.940	0.058	0.049	0.981	0.950	0.915	0.940

RMSEA = root mean square error of approximation; SRMR = standardized root mean square residual, GFI = goodness-of-fit index; AGFI = adjusted goodness-of-fit index; NFI = normed fit index; CFI = comparative fit index.

**Table 4:** Direct, indirect, and total effects of factors on conflict between older patients and family members during hospitalization.

		95% CI		
	Standardized Estimate	Lower	Upper	*p*
Direct Effect on Conflict				
Spouse provided most support--->Depression symptoms	−0.119	−0.191	−0.047	0.003
FSU immigrants--->Depression symptoms	0.124	0.049	0.199	0.002
Independence in ADL--->Depression symptoms	−0.368	−0.447	−0.286	<0.001
Israeli Arabs--->Emotional support	0.089	0.015	0.16	0.019
Spouse provided most support--->Instrumental care	−0.076	−0.136	−0.017	0.014
Independence in ADL--->Instrumental care	−0.565	−0.642	−0.486	<0.001
FSU immigrants--->Number of visitors	−0.147	−0.212	−0.078	<0.001
Israeli Arabs--->Number of visitors	0.313	0.213	0.41	<0.001
Depression symptoms--->Emotional support	−0.094	−0.173	−0.014	0.024
Israeli Arabs--->Instrumental care	0.08	0.005	0.157	0.037
Independence in ADL--->Conflict	−0.023	−0.126	0.078	0.659
Depression symptoms--->Conflict	0.032	−0.054	0.119	0.483
Instrumental care--->Conflict	0.146	0.048	0.248	0.003
Emotional support--->Conflict	−0.105	−0.186	−0.027	0.007
Number of visitors--->Conflict	0.125	0.029	0.215	0.011
Spouse provided most support--->Conflict	0.159	0.074	0.238	<0.001
Israeli Arabs--->Conflict	0.046	−0.039	0.135	0.309
FSU immigrants--->Conflict	0.106	0.019	0.193	0.016
Indirect Effect on Conflict				
Israeli Arabs--->Instrumental care--->Conflict	0.014	0.001	0.034	0.04
Israeli Arabs--->Number of visitors--->Conflict	0.048	0.011	0.089	0.011
Israeli Arabs--->Emotional support--->Conflict	−0.012	−0.026	−0.001	0.026
FSU immigrants--->Number of visitors--->Conflict	−0.02	−0.036	−0.004	0.011
FSU immigrants--->Depression symptoms--->Emotional support--->Conflict	0.001	0	0.003	0.031
Spouse provided most support--->Instrumental--->Conflict	−0.011	−0.024	−0.001	0.017
Spouse provided most support--->Depression symptoms--->Emotional support--->Conflict	−0.001	−0.003	0	0.034
Independence in ADL--->Instrumental--->Conflict	−0.002	−0.004	−0.001	0.003
Depression symptoms--->Emotional support--->Conflict	0.01	0.001	0.024	0.031
Total Effect on Conflict				
Independence in ADL--->Conflict	−0.122	−0.206	−0.038	0.007
Depression symptoms--->Conflict	0.042	−0.045	0.129	0.341
Instrumental care--->Conflict	0.146	0.048	0.248	0.003
Emotional support--->Conflict	−0.105	−0.186	−0.027	0.007
Number of visitors--->Conflict	0.125	0.029	0.215	0.011
Spouse provided most support--->Conflict	0.142	0.059	0.222	0.001
Israeli Arabs--->Conflict	0.087	0.002	0.175	0.045
FSU immigrants--->Conflict	0.093	0.008	0.179	0.033

## Data Availability

The data presented in this study are available on request from the corresponding author due to ethical considerations.
